# Chromatography-Free Analysis of Mixtures Using a Two-Dimensional Mass Spectrometry (2DMS)-Enabled Quadrupole Time-of-Flight (QToF) Analyzer

**DOI:** 10.1021/acs.analchem.6c00486

**Published:** 2026-04-23

**Authors:** Steven Wright, Nathan Cassidy, Alex Colburn, Peter B. O’ Connor

**Affiliations:** † Verdel Instruments Ltd, 154k Brook Drive, Milton Park, Abingdon, Oxfordshire OX14 4SD, U.K.; ‡ Department of Chemistry, University of Warwick, Coventry CV4 7AL, U.K.

## Abstract

Two-dimensional mass spectrometry (2DMS) is a powerful data-independent analysis (DIA) technique that allows mixtures to be analyzed without chromatographic separation of the components. Until now, high-resolution 2DMS has only been possible using Fourier Transform Ion Cyclotron Resonance (FTICR) instruments, which are impractical for most laboratories. Herein, we present the first experimental demonstration of 2DMS using a quadrupole time-of-flight (QToF) mass spectrometer. These instruments are commonplace, but offer high resolution and the ability to assign peaks in the 2D spectrum based on accurate mass. The key development is the application of stored waveform ion radius modulation (SWIM) to ions trapped in the quadrupole prior to mass analysis by the ToF analyzer. SWIM is an encoding technique that uses broadband dipolar excitation and a radius-dependent fragmentation method to modulate precursor and product ion signals. We show how each component of a peptide mixture can be individually, yet simultaneously, sequenced using collision-induced dissociation (CID) and/or ultraviolet photodissociation (UVPD).

## Graphical abstract

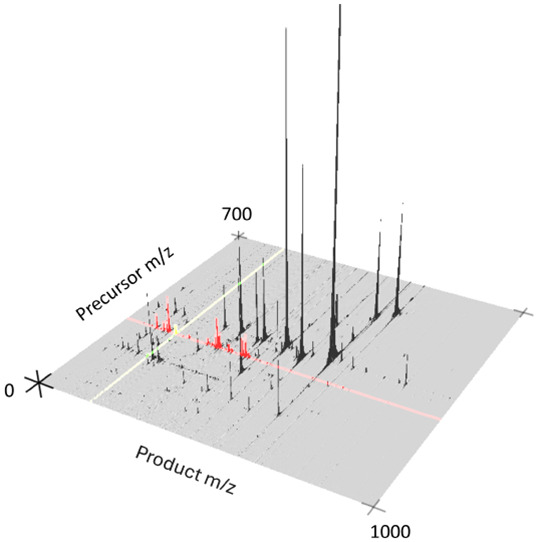

## Introduction

The ability to dissociate ions and determine their structures from the resulting fragments is a fundamental tool in mass spectrometry. Even in basic instruments, there is usually some capacity to perform collision induced dissociation (CID), the most widely used fragmentation method. More advanced instruments may include a plethora of other fragmentation techniques that have been thoroughly reviewed elsewhere.^1^ Typically, the components in a mixture are separated by chromatography before being presented to the mass spectrometer to ensure that the observed fragments are associated with a particular precursor. However, baseline separation of all the components in a complex mixture is often unattainable and chimeric spectra containing fragments from multiple coeluting analytes occur frequently. In selected reaction monitoring (SRM), precursors in a preprogrammed list are isolated using a quadrupole mass filter, but this requires prior knowledge of the sample composition and is only suitable for targeted analyses. Precursor selection is determined on-the-fly in data-dependent analysis (DDA) by identifying the most intense peaks in a preliminary MS1 scan at the start of each measurement cycle. However, precursors with abundances below the trigger threshold are missed.

Data independent analysis (DIA) is a term used to describe a broad suite of techniques^2^ that attempt untargeted and unbiased acquisition of product ion spectra for every precursor present. Most use the first quadrupole in the system, Q1, as either a narrow or broadband precursor isolation filter. PAcIFIC and SONAR are examples of the former, using multiple injections and fast Q1 scanning, respectively, to allow the transmission window to slide over the full mass range of possible precursors.^2–4^ Techniques employing wide isolation windows rely on data processing to deconvolute the resulting chimeric product ion spectra. SWATH uses a query strategy based on retention times and library spectra,^5^ whereas in MS^E^, the collision energy is alternated between low and high values, and fragments are correlated with precursors by matching their chromatographic peak profiles.^6^ More recently, dynamic positioning of the isolation window based on ion mobility has been achieved using the PASEF technique.^7^

2DMS is a DIA technique that can simplify the untargeted analysis of complex mixtures by eliminating the need for chromatographic or quadrupole isolation. However, 2DMS can also complement chromatography. For example, a 2D spectrum could be used to generate a list of precursor and product ions to be analyzed in a targeted analysis, identify components missed in a DDA analysis, or deconvolute chimeric spectra. All components of a mixture are fragmented simultaneously and each of the resulting product ions is represented by a peak on a 3D surface, often represented as a 2D contour plot. Product ion peaks are characterized not only by their own *m*/*z* value (x-coordinate), but also by the associated precursor *m*/*z* value (y-coordinate).

The technique was originally demonstrated using Fourier Transform Ion Cyclotron Resonance (FTICR) instruments.^8,9^ Precursor and product ion signals are frequency-encoded using resonant excitation at the characteristic cyclotron frequency to modulate precursor ion orbital radii. This was first achieved using two excitation pulses separated by a variable delay^8,9^ and later by amplitude modulation.^10,11^ If a radius-dependent fragmentation technique is provided, the abundances of any product ions formed will modulate at the same frequency as the precursor ion. Applying the Fourier Transform to all the ion signals recovers the modulation frequencies and shows which ions are correlated and therefore, which fragments are derived from which precursors, even in mixtures. CID was used in the earliest work.^9–11^ The kinetic energy of precursor ions increases with orbital radius and hence, dissociation activated by collision with residual gas molecules becomes radius-dependent. More recently, various laser-based and electron-based options have emerged, including infrared multiphoton dissociation (IRMPD)^12^ and ultraviolet photodissociation (UVPD).^13^ Ion radius modulation changes the overlap between precursor trajectories and the laser beam, which is directed along the axis of the ICR cell, resulting in a concomitant modulation of the photofragment yield.^13–15^

Although a wide range of applications have been addressed by 2DMS,^13,16–21^ the impact of the technique has been limited by the high cost and scarcity of FTICR instruments. Nearly 10 years ago, it was recognized^22^ that in principle, QToF instruments, which are much more widely available, could also be adapted to perform 2DMS. In quadrupoles, ions oscillate as if trapped in a parabolic well,^23,24^ following trajectories referred to as secular motion. If the QToF quadrupole is converted to a linear ion trap, the orbital radii of trapped ions may be modulated in a similar way to the FTICR experiment, using resonant dipolar excitation^23,25^ at the characteristic secular frequencies.

In this paper, we present an experimental demonstration of 2DMS performed with a QToF for the first time. Both CID and UVPD at 213 nm have been implemented. This choice of wavelength is a compromise between photofragmentation efficiency and ease-of-use. Excimer lasers operating at 157 and 193 nm generate rich fragmentation spectra but atmospheric absorption at short wavelengths and the need for fluorine gas introduce practical difficulties. Solid state Nd:YAG lasers are more convenient and the fifth harmonic at 213 nm yields sufficient fragmentation of small molecules, peptides, and proteins to enable structural characterization.^26,27^

Frequency-encoding of the ion signals is achieved using stored waveform ion radius modulation (SWIM).^10,11^ SWIM is a derivative of the stored waveform inverse Fourier Transform (SWIFT) technique.^28–30^ In SWIFT, ion radial position can be preprogrammed with a defined, set excitation profile, which is calculated, inverse Fourier transformed, and stored in an arbitrary waveform generator for later application to ion radial excitation. In the most typical implementation of SWIFT, all ions other than a selected precursor are ejected from the trap by a broadband pulse. In the frequency domain, this pulse has a top-hat profile with a notch centered at the ion cyclotron frequency (FTICR) or secular frequency (quadrupole ion traps) of the precursor ion that is to remain in the trap. The isolated precursor can then be interrogated by a subsequent fragmentation step. Several variations have been devised to subtly manipulate and process trapped ions. The ion parking technique^31^ uses broadband pulses to radially excite but not eject ions with particular *m*/*z* values. These selected ions are thereby protected from ion–ion reactions occurring at the center of the trap. Similarly, in fragment ion protection (FIP) experiments,^32^ all fragments generated by photofragmentation are moved away from the laser path to prevent secondary reactions. However, the aforementioned methods are all essentially binary in nature—ions are either excited or not excited for the purposes of achieving separation. The SWIM technique is different in that the degree of excitation is sinusoidally varied in order to modulate the ion radial positions.

In recent years, other routes to 2DMS have been developed. A group at Purdue University^33,34^ has generated 2D spectra using a frequency tagging technique, while researchers at Imperial College^35,36^ have successfully pursued a method based on partial covariance. The Purdue instrument has produced spectra with steadily improving performance but is limited to nominal mass accuracy by the capabilities of the base mass spectrometer. The technique described here maintains the resolution and accurate mass capabilities of the QToF instrument in the product ion axis, allowing much higher confidence in peak assignments.

In the following sections, we first provide an overview of the encoding technique and modes of implementation. As the interplay between these modes is subtle, a simulation model has been developed. This is used to aid interpretation of the experimental data and provide insight into processes occurring in the quadrupole. Modification of a conventional QToF instrument to enable 2DMS is described in the Experimental Section. We then present experimental data to demonstrate modulation by broadband excitation, fragmentation by both CID and UVPD, and application of the technique to the analysis of a peptide mixture.

## Theory

### Frequency Encoding

A sequence of SWIM pulses was generated using the method first described by Ross et al.^10,11^ and later employed by van Agthoven and O’Connor.^22^ For a pulse with index *n*, the magnitude *M* at frequency *f* is given by eq 1.

1 $$M_{\left(f,n\right)}=\frac{1}{2}\left(1+\sin\left(n\pi \left(\frac{f-f_{min}}{f_{max}-f_{min}}\right)-\frac{\pi }{2}\right)\right)$$

The minimum and maximum secular frequencies are *f*_*min*_ and *f*_*max*_, respectively. To reduce the dynamic range and maximum voltage required of the DAC and amplifier, the pulse was spread in the time domain using the quadratic phase function^37,38^ given in eq 2.

2 $$\varphi_{\left(f\right)}=\frac{T{\left(f-f_{\min}\right)}^{2}}{\left(f_{max}-f_{min}\right)}$$

eq 3 is the frequency domain representation of the desired excitation, which is obtained by combining eqs 1 and 2.

3 $$PM_{\left(f,n\right)}=M_{\left(f,n\right)}\times e^{i\varphi_{\left(f\right)}}$$

Applying the inverse Fourier Transform and extracting the real component then yields the time domain pulse. In this work, *f*_*min*_ = 10 kHz and *f*_*max*_ = 500 kHz, respectively, which correspond to a precursor mass range of *m*/*z* 90–4500 (1 MHz trapping waveform with 240 V_peak–peak_ amplitude). The variable *T* determines the width of the pulse and was set to 10 ms, resulting in a maximum excitation amplitude of ∼1 V_zero‑peak_. Earlier work based on simulations suggested much narrower (380 μs pulse width) and more intense (70 V_zero‑peak_ amplitude) excitation waveforms,^22^ which are more challenging to generate in practice.

It is clear from eq 1 that as the pulse index is incremented, the amplitude at any given frequency follows a sinusoid. Hence, for a secular frequency, *f*_*s*_, the encoding frequency, *f*_*e*_, in the SWIM index domain is given by eq 4.

4 $$f_{e}=\frac{f_{s}-f_{min}}{2\left(f_{max}-f_{min}\right)}$$

The secular frequency is related to the frequency of the RF trapping waveform applied to the quadrupole rods, *f*_*trap*_, by *f*_*s*_ *= β x f*_*trap*_ */2*, where β is a parameter characterizing solutions of the Mathieu equations. The Dehmelt approximation, *β ≈ q/√2*, where *q* is the Mathieu stability parameter, may be applied if *q* < 0.4.^39^ As *q = zV*_*trap*_ */π* ^2^ *mr*_0_^2^ *f*_*trap*_^2^, where *m* and *z* are the ion mass and charge, respectively, *V*_*trap*_ is the zero-to-peak amplitude of the RF trapping waveform and *r*_0_ is the quadrupole field radius, it follows that the expected encoding frequency for any given precursor ion may be calculated using eq 5.

5 $$f_{e}=\frac{zV_{trap}}{4\sqrt{2}\pi^{2}mr^{2}f_{trap}\left(f_{max}-f_{min}\right)}-\frac{f_{min}}{2\left(f_{max}-f_{min}\right)}$$

In common with the original implementation of SWIM using FTICR,^10,11^ there is a linear relationship between encoding frequency and *z/m*. Koizumi et al.^40^ have developed an expression for *m*/*z* in terms of *f*_*s*_ that is accurate for all stable values of *q* and therefore avoids the *q* < 0.4 restriction of the Dehmelt approximation. Combining this expression with eq 4 yields eq 6, which may be used to convert from *f*_*e*_ to *m*/*z* when high *q* (low *m*/*z*) precursor ions are present.

6 $$\frac{m}{z}=\frac{\pi V_{trap}}{16r^{2}f_{trap}^{2}}{\{\frac{3}{2}[35-\sqrt{125-70\left(1-\cos\left(2\pi \frac{2f_{e}\left(f_{\max}-f_{\min}\right)+f_{\min}}{f_{trap}}\right)\right)}]\}}^{-0.5}$$

### Modes of Operation

A simplified representation of the signal encoding process is shown in Figure 1a. The orbital radius starting from n = 0 (no excitation) is modulated sinusoidally as *n* increments. If a Gaussian laser beam is directed along the axis of the trap, the probability of photofragmentation is highest at low orbital radius, where the beam is most intense, and lowest at high radius, where the beam is less intense. While this mirrors the interpretation of SWIM applied to FTICR, in which coherent ion packets follow circular trajectories of well-defined radius, ion populations in quadrupoles are characterized as ion clouds.^41^ Consequently, the application of SWIM to quadrupoles is better described as a modulation of the ion cloud’s average radius. Modulation of in-trap CID is similar except that the relationship between orbital radius and fragmentation probability is inverted; ions excited to higher radius have more kinetic energy and are therefore more likely to undergo energetic collisions with residual gas. For both UVPD and CID, the precursor and associated product ion intensities are modulated at the same frequency, but the waveforms are 180° out-of-phase.

**Figure 1:**
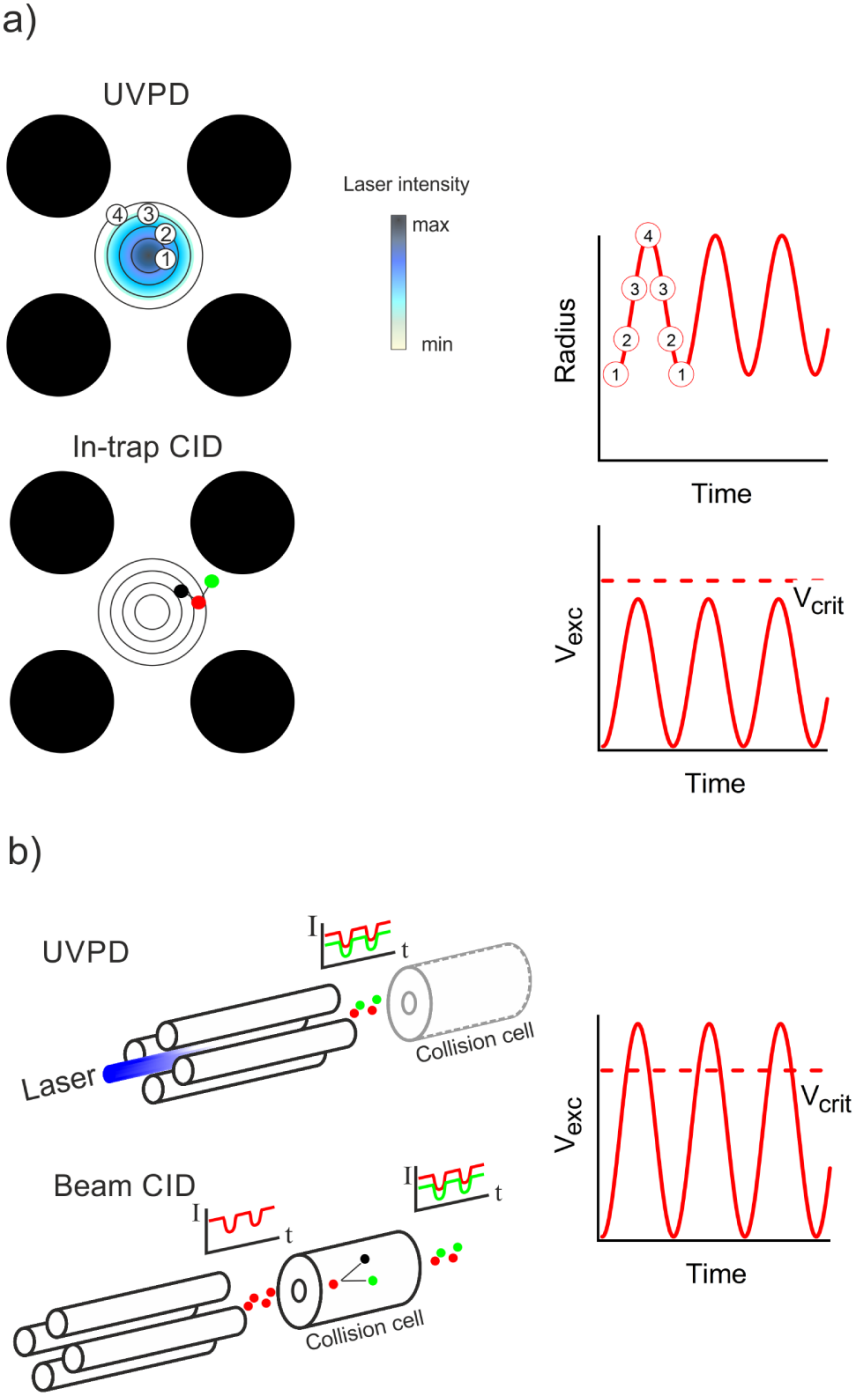
Modes of operation. (a) In the flux-constant mode, the ion orbital radius is modulated sinusoidally by a dipole excitation of magnitude V_exc_. Fragmentation is modulated by the changing irradiance within a Gaussian laser beam (UVPD) or varying ion kinetic energy (in-trap CID). (b) In flux-periodic mode, a portion of the ion cloud is lost when the excitation amplitude exceeds V_crit_. Fragment yields from UVPD or beam CID follow the modulation of the precursor abundance.

It has been assumed above that there are no ion losses and the total number of ions entering and leaving the trap are equal. This mode of operation will be referred to below as the flux-constant mode. However, as the magnitude of the radial excitation is increased, some portion of the ion cloud will be unable to pass through the exit aperture during the trap emptying phase (discharging on the exit lens instead). At sufficiently high excitation, ions will be lost by ejection from the trap, as noted in earlier simulations.^22^ Excitation is at a maximum once per modulation cycle and so it is anticipated that there will be corresponding periodic dips in the total number of transmitted ions due to these exclusion mechanisms, as indicated in Figure 1b. Hence, even in the absence of in-trap fragmentation, precursor ion signals will be modulated at the encoding frequencies prescribed by eq 5. This mode will be described below as the flux-periodic mode.

The flux-periodic mode opens up an alternative fragmentation method, using beam CID rather than in-trap CID. In beam CID, the precursor modulation and fragmentation steps are separated in both time and space. Ions are accelerated by a potential difference (typically 10–200 V) between the quadrupole and a downstream multipole contained within a collision cell. A gas bleed into the collision cell jacket provides an elevated pressure of N_2_. A fraction of the precursors entering the collision cell undergo fragmentation. Consequently, the product ion yields are expected to follow the precursor modulation with the same frequency and phase.

### Simulations

Radial excitation of the ion cloud by SWIM and subsequent fragmentation by UVPD were modeled using SIMION 8.2 (Scientific Instrument Services, Ringoes, NJ, USA). A full description of the simulation strategy and a code listing is provided in the Supporting Information (SI). Briefly, a two-step approach has been adopted. First, a realistic initial ion cloud was generated by injecting ions into a full ion optics model and subsequently extracting the ion positions and velocities after a period of collisional cooling. This distribution was then transferred to a high-definition model of just the quadrupole section for simulation of radial excitation during application of a SWIM pulse. A hard sphere collision model was used to simulate collisions with background gas. The probability of photofragmentation was calculated by considering the path-averaged photon exposure during one secular orbit through the Gaussian laser beam. The fate of an individual precursor ion was determined using the Monte Carlo method. Any fragment produced received a portion of the photon energy with a randomized velocity vector being added in a center-of-mass frame of reference. The output for each SWIM pulse in the sequence is a tally of the number of fragments and surviving precursor ions.

## Experimental Section

### Materials

Polymyxin B1 solution (1 mg/mL, analytical standard), bacitracin A (powder, 57%), substance P acetate salt hydrate (powder, 97%), melittin from honey bee venom (powder, 93%), and reserpine standard for LC-MS (5 μg/mL) were all purchased from Sigma-Aldrich (UK). HPLC-grade water, acetonitrile, and formic acid were also purchased from Sigma-Aldrich (UK). ESI-L low concentration tuning mix was obtained from Agilent Technologies (USA).

### 2DMS-Enabled QToF Mass Spectrometer

A Bruker MaXis II (ETD) QToF mass spectrometer (Bruker Daltonik GmbH, Bremen, Germany) was modified to allow operation of the quadrupole as an ion trap with dipolar excitation and coaxial laser irradiation. A replacement quadrupole chamber lid with multiple vacuum feedthroughs allowed existing RF and DC supplies to be bypassed. A bespoke 1 MHz RF generator comprising an amplifier circuit and a 1:25 step-up transformer was used to supply the 240 V_peak–peak_ trapping waveform. Dipolar excitation requires waveforms of equal magnitude but inverted polarity to be applied to two opposing rods. The straps that coupled opposing rods were removed and the in-vacuum network of resistors and capacitors was modified to allow the necessary electrical isolation of the four main quadrupole rods.

SWIM pulses were calculated in advance and stored as a single binary file. During data acquisition, individual SWIM pulse definition files were sequentially transferred to a PXIe 5450 arbitrary waveform generator (National Instruments), which generates normal and polarity-inverted pulses at 25 Msamples/s. The pulses were amplified and superimposed on a DC offset before being applied to an opposing rod pair via the secondary winding of the RF transformer. A PXI 6733 analogue output card (National Instruments) generated gating DC potentials that were amplified and applied to lenses upstream and downstream of the quadrupole. Ions were trapped in the quadrupole by setting the lens voltages to 25 V higher than the quadrupole rod bias. Each SWIM pulse has a duration of 20 ms but the excitation is mainly localized between 5 and 13 ms. At t = 0, the trap is filled by opening the entrance gate for 0.5–5 ms with the exit lens closed. At t = 18 ms, ions are released by opening the exit gate for 2 ms. For UVPD, laser trigger pulses are applied between t = 13 and 18 ms. The 20 ms trap-and-release cycle was repeated 50 times for each SWIM index and summed ToF spectra were saved at a matching rate of 1 Hz. A sequence of 1024 SWIM pulses was typically used and the acquisition time for a 2D spectrum was therefore 17 min. From eq 4, it is clear that the encoding frequencies will range from 0 to 0.5 Hz, where the upper limit represents the Nyquist limit for a 1 Hz sampling rate.

The experiment was controlled by Python code executed in a Visual Studio environment. The PXIe 5450 and PXI 6733 cards were mounted in an ADLINK PXES-2314T (ADLINK Technology Inc., Taiwan) chassis and controlled using NI-FGEN and NI-DAQmx drivers via a Thunderbolt interface. The Bruker MaXis mass spectrometer operated independently and saved data locally after receiving a start trigger from the PXI 6733 card. In postprocessing, the raw data files were unpacked using the Baf2SQL library provided by Bruker.

UVPD of trapped ions requires propagation of the laser beam coaxial with the ion optical axis. Access can be achieved by incorporating a 90° bend or dogleg in the ion path.^42^ Alternatively, the beam can be passed between the plates of the pusher stack in the ToF analyzer,^43^ which is the approach used in this work. A Crylas eMOPA 213–20 laser (Crylas GmbH, Berlin, Germany) and two steering mirrors were mounted on an external optics table. The laser beam entered the ToF analyzer through a CaF_2_ window and was then deflected through the pusher electrode stack and thereafter through the collision cell, quadrupole trap, and ion transfer optics using an in-vacuum mirror mounted at 45° between the pusher and the detector. The final collision cell lens was drilled out from 0.5 to 2 mm to accommodate the full width of the laser beam, which results in an increased but tolerable pressure in the ToF analyzer. Profiling of the laser beam in the quadrupole chamber (with the quadrupole removed) showed that the beam was approximately Gaussian with a fwhm width of 0.6 mm and a pulse energy of 25 μJ. The beam is eventually dumped near the capillary inlet in the first vacuum stage. Irradiation and possible photofragmentation of ions upstream of the trap in the ion transfer optics is unavoidable. However, these fragments are produced before application of SWIM and can therefore be distinguished from fragments generated in-trap (they appear on the autocorrelation line, described below).

## Results and Discussion

### Signal Modulation

Modulation of the ion signals by SWIM is demonstrated in Figure 2a. Baseline signals for Agilent tune mix were recorded during the first 130 s with the SWIM pulse amplitude set to zero. While the SWIM pulse amplitude was reset to approximately 2 V_peak–peak_, no ions were transmitted as the trapping lenses default to their closed states (130–190 s). On resumption of the trap-and-release cycle at 190 s, the ion signals modulated with a frequency inversely proportional to their *m*/*z* value, as required by eq 5. The flux-periodic mode is the only mechanism involved here as there is no fragmentation of the tune mix components (the laser is off and the collision energy is set at a low value).

**Figure 2:**
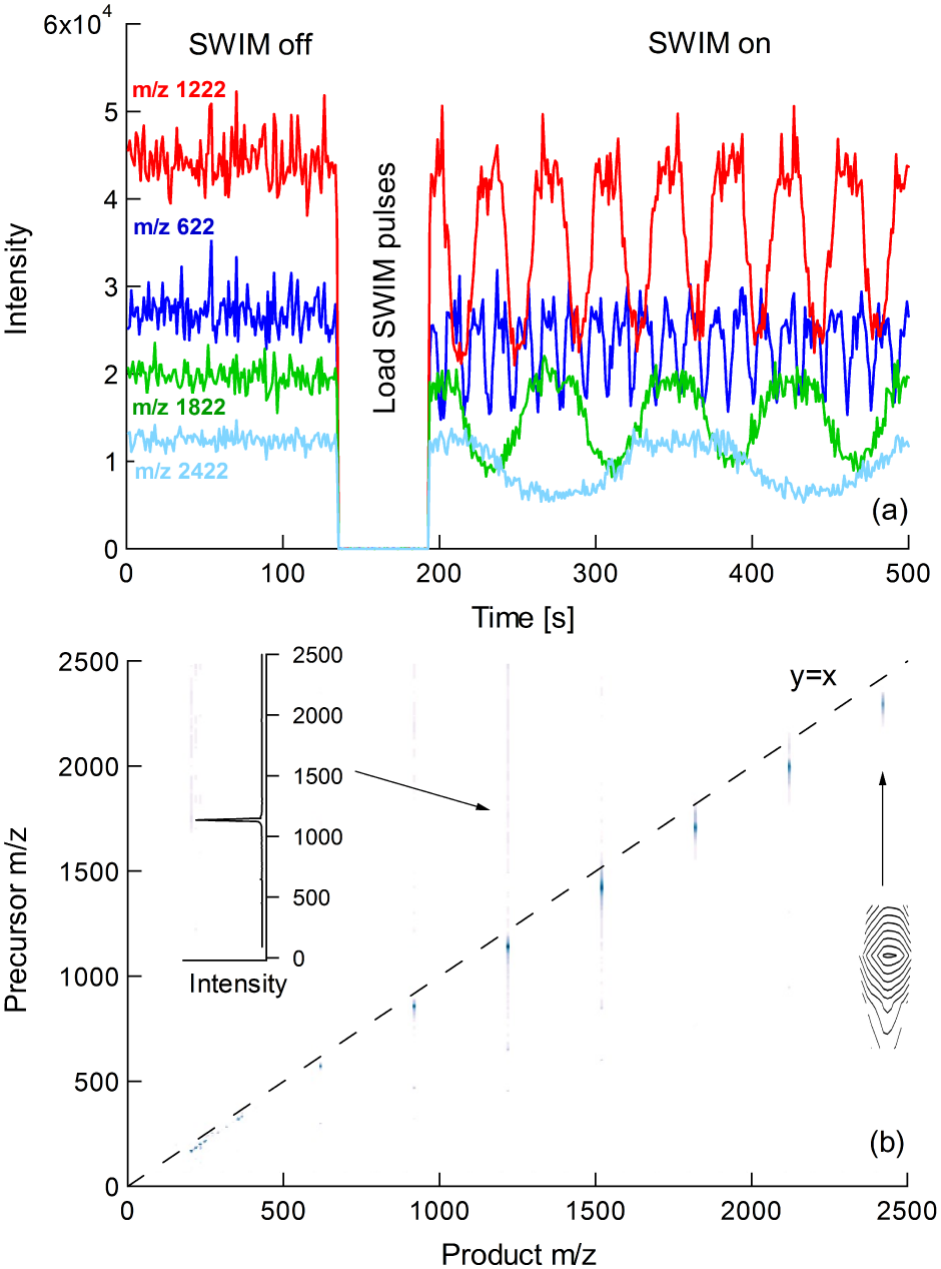
(a). Time domain data for components of Agilent tune mix. Baseline signals were recorded for the first 130 s. A SWIM pulse sequence was started at 190 s with pulse amplitudes of ∼2 V_peak–peak_. (b) Corresponding 2D mass spectrum presented as a contour map. As there is no fragmentation, all peaks lie on the autocorrelation line. The inset graph shows a vertical slice at *m*/*z* 1222.

Recording a data set for *n* = 1–1024 and zero-filling once generates an array of 2048 rows and approximately 10^6^ columns, where each column represents the time domain signal for an individual *m*/*z* bin in the ToF spectrum. Conversion to the frequency domain is achieved by applying the Fourier Transform to the data in each column, yielding 1024 rows of evenly distributed, real modulation frequencies. Finally, the precursor encoding frequency is re-expressed as precursor *m*/*z* by inverting eq 5 so that it is in the form *(m/z)*_*precursor*_ *= k*_1_ */(f*_*e*_ *+k*_0_), allowing the data set to be plotted as a 2D mass spectrum. As a consequence of the reciprocal relationship between encoding frequency and *m*/*z*, the bin widths are smaller at low *m*/*z* than at high *m*/*z*.

Figure 2b shows a 2D mass spectrum for Agilent tune mix. The data is presented as a color-indexed contour map after thresholding to remove most of the noise floor. As there is no fragmentation, all peaks lie on the diagonal autocorrelation line (precursor *m*/*z* = product *m*/*z*). There is a small deviation from y = x because eq 5 is based on an assumed ideal quadrupole field and an approximation for *β.* eq 5 also involves *V*_*RF*_, which is not easily measured accurately. In practice, the whole data set may be conveniently recalibrated by plotting *(m/z)*_*product*_ against *(m/z)*_*precursor*_ for all the peaks on the autocorrelation line, calculating a linear least-squares regression line, and then applying a modified calibration equation of the form *(m/z)*_*precursor*_ *= c*_1_ *(k*_1_ */(f*_*e*_ *+k*_0_ *))+c*_0_. Better accuracy can be achieved with a quadratic or cubic fit.

Some vertical streaking^44^ is apparent, particularly for the most intense peak at *m*/*z* 1222. This is a consequence of the broad-spectrum noise present in the time domain data. It is clear from the left-hand side (0–130s) of Figure 2a that the noise amplitude is proportional to the signal intensity. Any attempt to discriminate against this noise for *m*/*z* 1222 by setting the threshold at a high level risks eliminating the *m*/*z* 2422 data, whose SWIM-induced modulation is of a similar amplitude. Taking a vertical slice at *m*/*z* 1222 (shown inset) illustrates that despite this streaking in the contour map presentation, the peak is intense and the noise floor is low.

Operation in the flux-constant CID mode is demonstrated in Figure 3 using substance P as a model analyte. The [M+3H]^3+^ precursor dissociates to give b_10_^2+^ fragments under mild CID conditions. A mass spectrum recorded during broadband SWIM pulse excitation is shown in Figure 3a and extracted peak intensities for the precursor and product ions are plotted in Figure 3b. RF amplitudes and DC gradients in the upstream and downstream ion optics were reduced to minimize background fragmentation outside the trap, but some residual baseline fragmentation remains. Figure 3b proves the fundamental requirement for 2DMS; the precursor and associated product ion signals modulate at the same frequency. A link has therefore been established between the two. The modulation frequency is 0.101 ± 0.002 Hz, which is in excellent agreement with the theoretical prediction from eq 5 of 0.102 Hz. In accordance with the flux-constant model, the product ion intensity modulation is phase-shifted by 180°. The modulations are periodic but quite asymmetric. Fragmentation only occurs above a threshold amplitude during resonant dipolar excitation,^25^ and this is the likely cause of the asymmetry; fragments are formed only during the portion of the radius modulation cycle that lies above the threshold excitation rather than varying sinusoidally. The inset in Figure 3a shows the fragment yield increasing as the SWIM pulse is increased from 0 to 1.4 V_peak–peak_.

**Figure 3:**
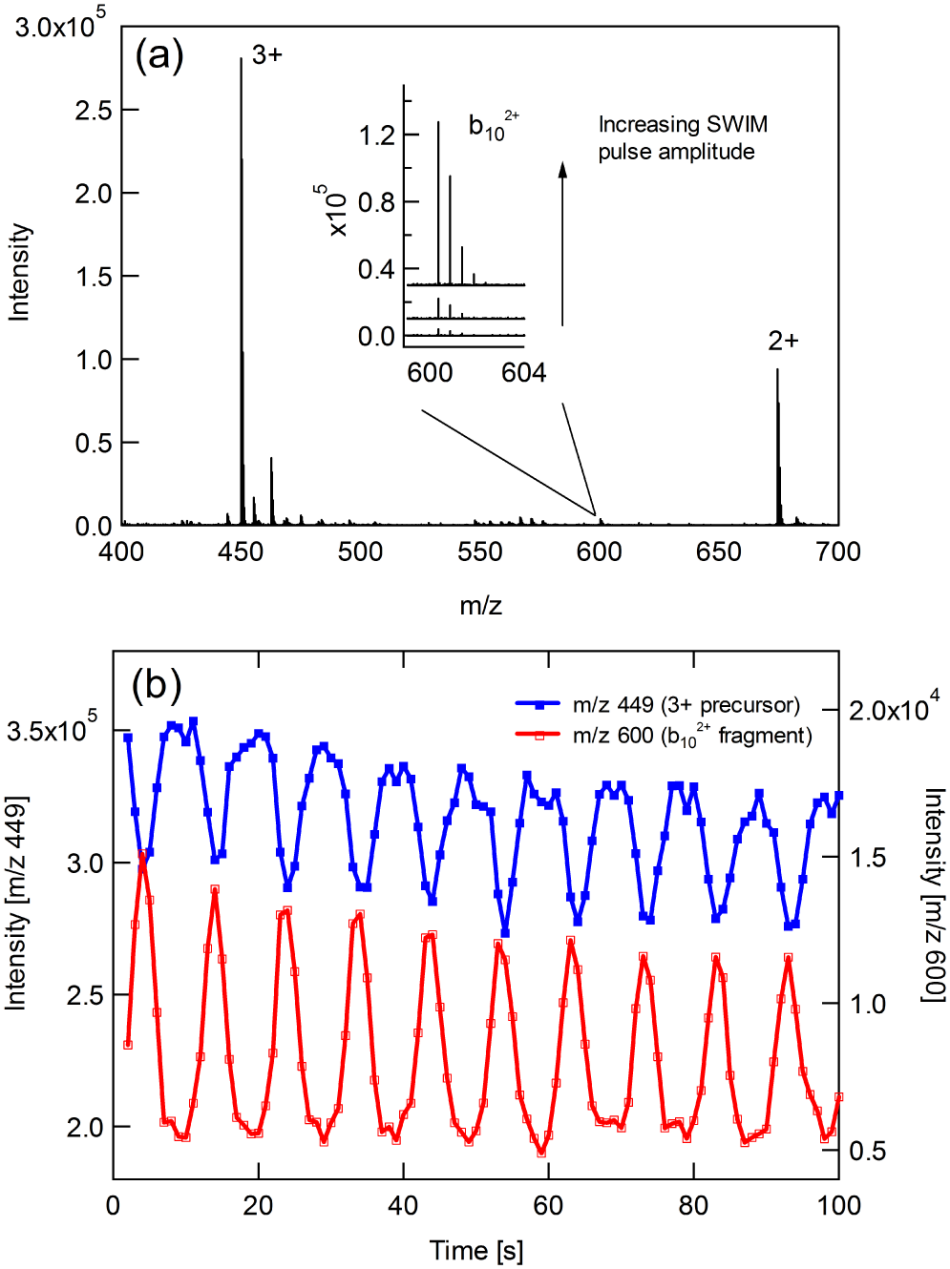
(a). Mass spectrum of substance P during SWIM excitation. (b) Extracted peak heights for the [M+3H]^3+^ precursor and b_10_^2+^ fragment. The modulation frequencies are identical, but the waveforms are phase-shifted by 180°.

Figure 4 demonstrates modulation of UVPD using SWIM. The model analyte here is reserpine, which yields a prominent fragment at *m*/*z* 195 following irradiation at 213 nm. In Figure 4a, the excitation amplitude is set at a low value to favor the flux-constant mode. The precursor and fragment intensities are once again modulated at the same frequency with a phase shift of 180°. As the precursor *m*/*z* is higher than in the previous example, the modulation frequency is lower. There is some distortion at *t* < 5s because the laser takes several seconds to warm-up and reach full power. The simulations shown in Figure 4b are in excellent agreement with the experimental data. The modulations have the same frequency, waveform shape, and phase relationship as the experimental data.

**Figure 4:**
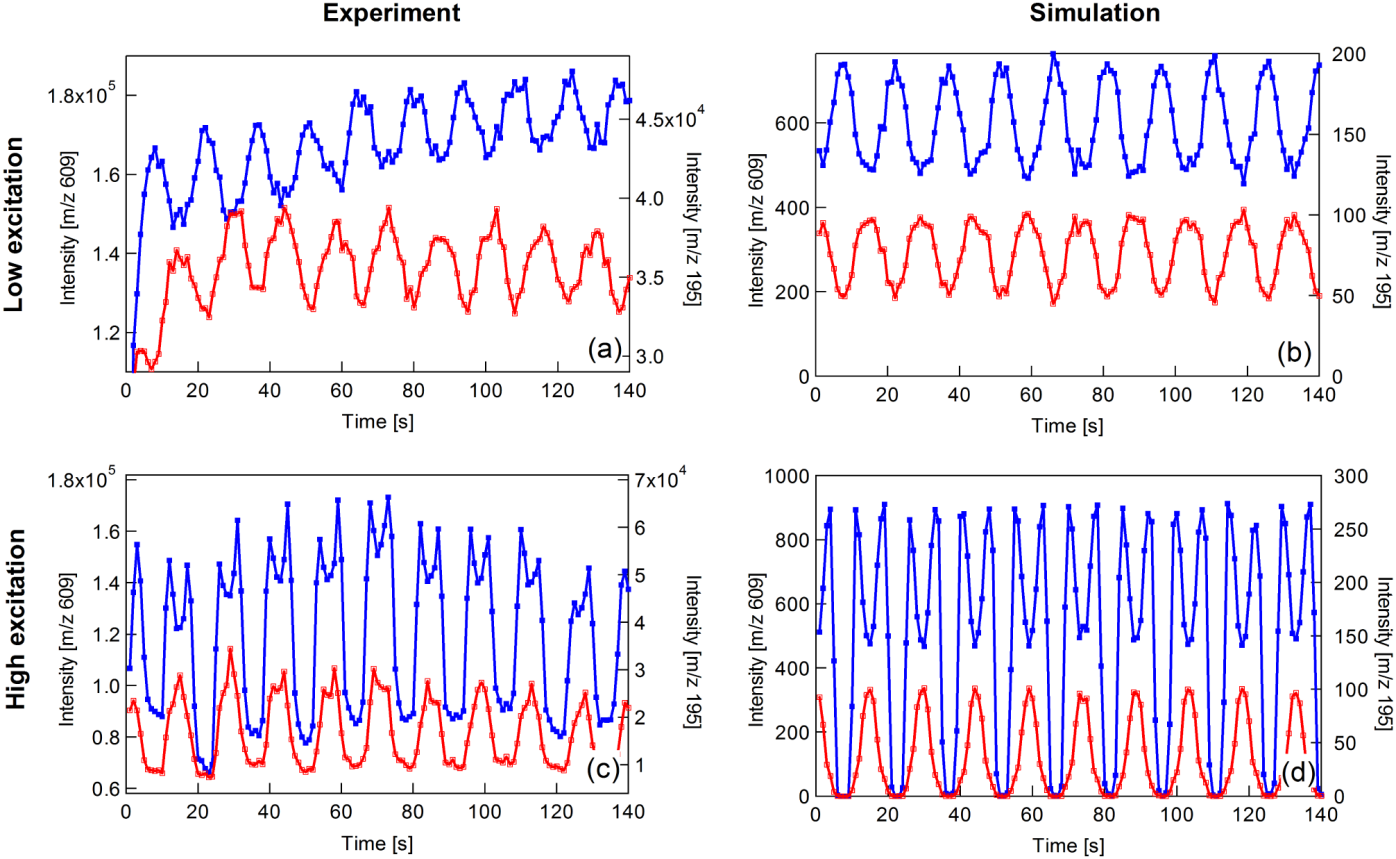
Extracted peak heights for reserpine (blue) and its *m*/*z* 195 fragment (red) during UVPD. The upper panels show operation in the flux-constant mode (SWIM pulse amplitude ∼0.5 V_peak–peak_) whereas the lower panels show mixed-mode operation (SWIM pulse amplitude ∼2 V_peak–peak_).

Increasing the pulse amplitude to ∼2 V_peak–peak_ results in a transition to the flux-periodic mode. Figure 4c and d show that both the precursor and product ion intensities are simultaneously depleted once per cycle. However, an artifact of the weaker flux-constant mode is superimposed on the waveform. There is a small dip at the peak of each precursor cycle due to residual flux-constant mode modulation. At the center of each dip, radial excitation is at a minimum and overlap with the laser beam is maximized, resulting in increased depletion of the precursor population. This interpretation is reinforced by the simulations, which reproduce the mixed-mode modulations without any modification of the model or its parameters, other than the excitation amplitude.

The double dip in each cycle introduces a modulation at *2f*_*e*_. However, this is generally weak compared with the fundamental mode at *f*_*e*_. It is worth noting in this figure that regardless of any deviation from purely sinusoidal modulation, the waveform is still periodic at the intended frequency, so the Fourier transforms of these ion signals will generate a fundamental that is the encoding frequency of the precursor. The asymmetric waveforms evident in Figures 2 and 3 are other examples of distortions that will generate additional harmonics. Deviations from a purely quadrupolar trapping field may also contribute. While additional peaks corresponding to the harmonics may be seen in the 2D spectrum, these appear at integer multiples of the encoding frequency and are consequently easily recognized.

Displacing the laser beam in the simulation model shows that alignment of the laser with respect to the ion cloud has a significant but asymmetric effect on the flux-constant mode. The asymmetry is due to the direction of radial excitation. Excitation to circular ion trajectories, as shown in Figure 1a, has been reported,^23^ but the dipolar excitation across one rod pair employed in the present work results in radial excitation and elongation of the ion cloud predominantly in one *x*-axis. Higher order field components and gas collisions can cause some coupling with the orthogonal axis, however.^45^ Modulations corresponding to lateral laser beam displacements along the x- and *y*-axes are shown in Figure S1. The rod pair receiving the dipolar excitation is aligned with the *x*-axis. The simulations show that *x*-axis displacements cause the ion cloud to expand both into and away from the most intense region of the laser profile. There is a null point where the net effect eliminates the signal modulations, and a phase reversal at bigger displacements. This suggests that the first harmonic contribution to mixed-mode operation could be reduced, although precise control of the laser yaw, pitch, and lateral offset would be required.

### Application to Peptide Mixtures

In this section, application of 2DMS to the analysis of a polymyxin, melittin, bacitracin, and substance P mixture will be presented. These peptides, which are all present with multiple charge states, fragment readily when subjected to low-energy CID or UVPD at 213 nm.

Figure 5a and b show 2D mass spectra for this mixture obtained using CID and UVPD, respectively. In both cases, the excitation amplitude is set at 2 V_peak‑to‑peak_ to favor the flux-periodic mode, as this yields stronger modulations and more intense peaks in the 2D mass spectrum. For the CID experiment, a solution was prepared with polymyxin and substance P at 333 ng/mL, and bacitracin and melittin at 667 ng/mL. The concentrations were 5-fold higher for the UVPD experiment to improve the signal-to-noise ratio and compensate for the generally lower level of fragmentation compared with CID. As in Figure 2b, each spot in the 2D spectrum is a peak represented by several closely nested contours. Every peak appearing on the autocorrelation line is a precursor. A section through the plot along the autocorrelation line may be regarded as a precursor mass spectrum (equivalent to an MS1 scan), although peak intensities on the autocorrelation line do not only reflect abundance; they are also affected by fragmentation efficiency. In addition to the four peptides (in multiple charge states), there are numerous other generally low-intensity contaminant precursors arising from impurities in the as-purchased peptide stock and solvents as well as leachates, all of which are ubiquitous in ESI-MS. In contrast to Figure 2b, there are very many peaks that are not on the autocorrelation line in Figure 5a and b. Each one represents a product ion. These fragment peaks are arranged in horizontal lines. All the fragments lying on a particular horizontal line belong to the same precursor. A peak corresponding to an intact precursor ion is found at the intersection of each horizontal line with the autocorrelation line. There is a very weak first harmonic autocorrelation line (at approximately y = 0.5x) and associated field of secondary product ion peaks in the bottom half of Figure 5a that arise from the modulation distortions mentioned above.

**Figure 5:**
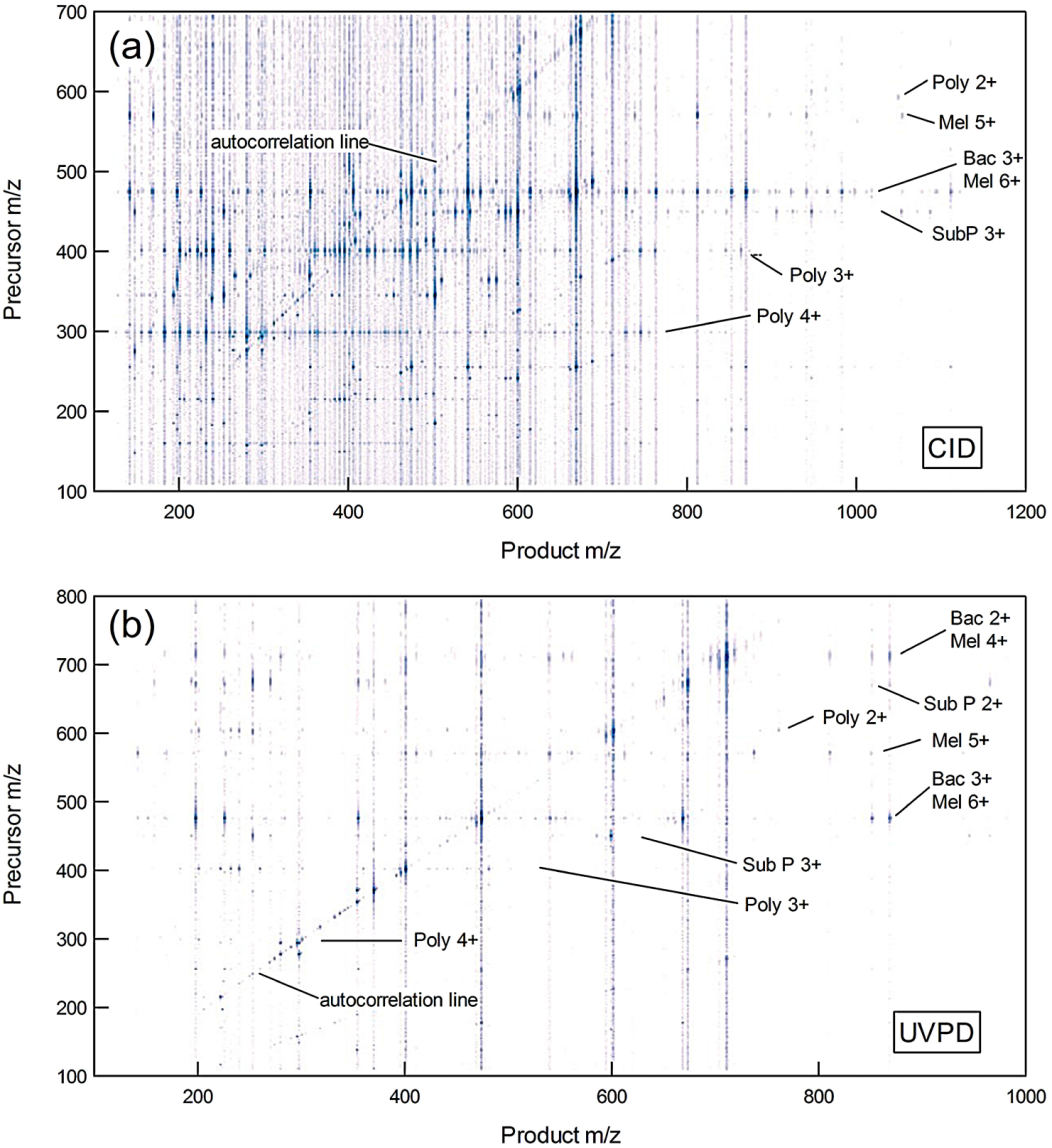
2D mass spectra of a bacitracin (Bac), melittin (Mel), substance P (Sub P), and polymyxin (Poly) mixture obtained using (a) CID and (b) UVPD.

The precursor axes have been calibrated using peaks lying on the autocorrelation lines. Figure S2 shows that a better first-pass conversion from *f*_*e*_ to *m*/*z* is achieved using eq 6 rather than eq 5 as the Dehmelt approximation leads to curvature below *m*/*z* 400. The remaining residual error is removed using a quadratic fit. Figure S3 shows that the peptide precursor peaks are located with a maximum *y*-axis error of −0.79 *m*/*z*. The widths of the precursor peaks in the y-coordinate are also plotted in Figure S3 for both the peptide mixture and Agilent tune mix. Despite the increase in peak width with *m*/*z*, the peak position can be determined with an accuracy much smaller than the *m*/*z* bin width by calculating a centroid value. Figure S3 indicates that there is only a weak dependence on peak width over most of the mass range.

A particular product ion may be produced by fragmentation of more than one precursor (for example, when a group of analytes share the same structural motif or if multiple charge states of the same analyte are present). When this is the case, a vertical slice at the product ion’s *m*/*z* value is equivalent to a precursor scan in conventional tandem MS. Figure S4 shows that the [T-DAB]^+^ fragment is produced by all three polymyxin charge states. Here, the peaks are well-resolved, and their positions can be accurately determined from a centroid calculation as above. However, for more closely spaced peaks, resolution in the precursor axis becomes limited by the peak width. Figure S3 indicates that the resolving power is *M/ΔM* ≈ 100.

The mass accuracy and resolution in the product axis are very much higher as the performance of the ToF analyzer is retained in this dimension. Figure S5 shows a horizontal slice through the polymyxin [M + H]^3+^ isotope cluster; the mass accuracy is 2.5 ppm and the peak width is 0.01 *m*/*z* (fwhm).

At y ≈ *m*/*z* 475 there are two overlapping series of peaks arising from the bacitracin [M+3H]^3+^ and melittin [M+6H]^6+^ precursors, which differ by only 0.2 *m*/*z*. However, the analysis is not blind to the presence of these two components. A 1D spectrum of the mixture recorded at low collision energy or with the laser switched off (so that no fragments are produced) reveals all the precursors present with the full resolution and mass accuracy of the ToF analyzer. Precursors that will not be vertically fully separated in the 2D spectrum are easily flagged. In this particular example, melittin can be separately sequenced and identified using the [M+5H]^5+^ precursor at *m*/*z* 570, and its fragments then eliminated from the overlapping series at *m*/z 475, allowing bacitracin to be sequenced and identified. Figure 6 shows the *y*-axis positions of all peaks that may be attributed to either the melittin [M+6H]^6+^ or bacitracin [M+3H]^3+^ monoisotopic precursor in Figure 5a. These peak positions have been extracted by taking a 7-point centroid, centered on the peak maximum. The means of the distributions are within 0.3 *m*/*z* of the expected accurate values of *m*/*z* 475.1329 and *m*/*z* 474.9236 for melittin [M+6H]^6+^ and bacitracin [M+3H]^3+^, respectively, which is similar to the mass accuracy achievable with a quadrupole mass filter. The standard deviation in both cases is 0.2 *m*/*z*, which does not allow full separation of the two distributions. Noise in the frequency domain is the primary contribution to the width of the distributions as this is summed together with the signal when calculating the centroid value.

**Figure 6:**
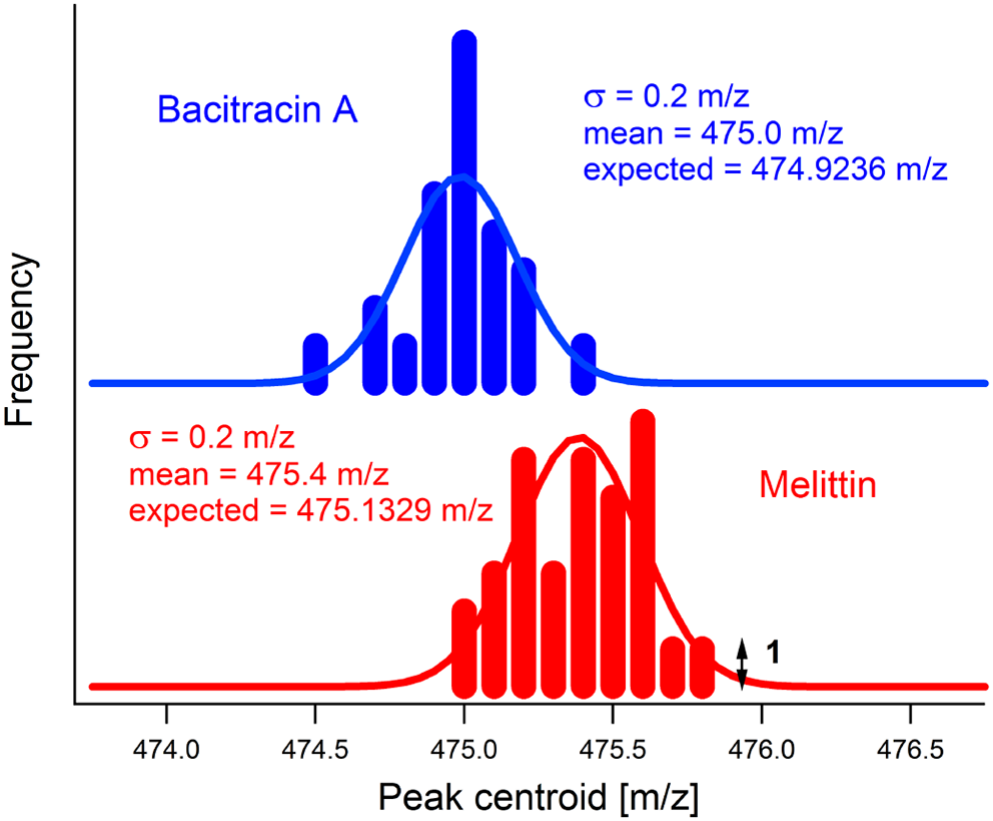
Peak position distributions (precursor *m*/*z* axis) for 34 peaks attributed to the melittin [M+6H]^6+^ precursor and 26 peaks attributed to the bacitracin [M+3H]^3+^ precursor. Normal distributions with the same means and standard deviations are also plotted.

Isolation windows are typically 1–4 *m*/*z* for DDA and 2–5 *m*/*z* for narrow-window DIA.^2,46^ Reducing the window width increases selectivity (at the expense of sensitivity) but eliminates or distorts the valuable information provided by the precursor isotope pattern.^47^ For both precursor and product ions, the isotopologue peaks allow the charge state to be assigned and increase the accuracy of elemental composition determinations. Consequently, ∼4 *m*/*z* windows are used to preserve the isotope distribution.^46^ Figure 6 demonstrates that 2DMS is capable of at least partial discrimination for precursors separated by only 0.2 *m*/*z*. Importantly, 2DMS preserves the isotope pattern. For example, three isotopologue peaks are seen in Figure S5, each separated by 0.333 *m*/*z* (confirming the +3 charge state assignment).

In Fourier theory, resolution is determined by the number of modulation steps within the experiment (1024 in this case). In principle, the resolution can be improved by increasing the number of modulation steps, or by narrowing the precursor *m*/*z* range of interest (by adjusting *f*_*min*_ and *f*_*max*_), although this is then a departure from data-independent analysis. In practice, the resolution achieved is dictated by acquisition time considerations and other practical limitations. A reduction in the trap-and-release cycle time and number of repeats thereof would allow the number of modulation steps to be increased without extending the acquisition time. A shorter excitation pulse is possible if the consequent increased amplitude (to maintain the same total impulse) and signal slew rate are within the capabilities of the amplifier. In the present work, 50 cycle repeats are required because of the large number of fragmentation channels. If the trap capacity is 10^5^ ions^23,48^ and there are 10^3^ fragmentation channels, then the average number of ions per fragment in a single fill is only 100. For applications that have a smaller number of fragmentation channels, it might be appropriate to use fewer repeats. As the number of modulation steps increases, the resolution will eventually be limited by the secular frequency line width, which will be affected by amplifier amplitude dither and collisions with background gas.^49^

The collision energy for the CID experiment was set at 20 eV, which results in extensive fragmentation of all four peptides. However, in general, the value chosen is inevitably a compromise between poor fragmentation of some analytes and over fragmentation of others, as all the components are analyzed simultaneously in the 2DMS experiment (in targeted tandem MS, the collision energy is typically optimized for each analyte). This is most likely to be problematic for mixtures comprising very dissimilar components, such as mixed chemical and biological samples.

Figure 7 shows a horizontal slice corresponding to the polymyxin [M+3H]^3+^ precursor at *m*/*z* 402 in Figure 5a. Polymyxin is a branched cyclic peptide with a complex fragmentation pattern.^50,51^ In general, cyclic peptide fragmentation begins with an initial ring-opening peptide bond cleavage and formation of a linear chain b-type ion.^52^ Charge-driven loss of neutral residues from the acylium terminus yields a series of further b ions that may be used to sequence the peptide. Overlapping series may be present if the initial cleavage can occur at multiple ring positions, and side-chain fragmentation should also be considered.^52^ In the case of polymyxin, three ring-opening cleavages have previously been identified.^50^ For clarity, only fragments arising from ring-opening at the Thr-Dab bond are explicitly labeled in Figure 7 while other assigned peaks are indicated with symbols. The maximum deviation from expected accurate *m*/*z* values is 4.6 ppm and the root-mean-square deviation for all assigned peaks is 1.8 ppm. The modified Roepstorff-Biemann nomenclature proposed by Ngoka and Gross has been used, which employs two additional descriptors to define the ring cleavage and numerical values to indicate side chain losses.^52^

**Figure 7:**
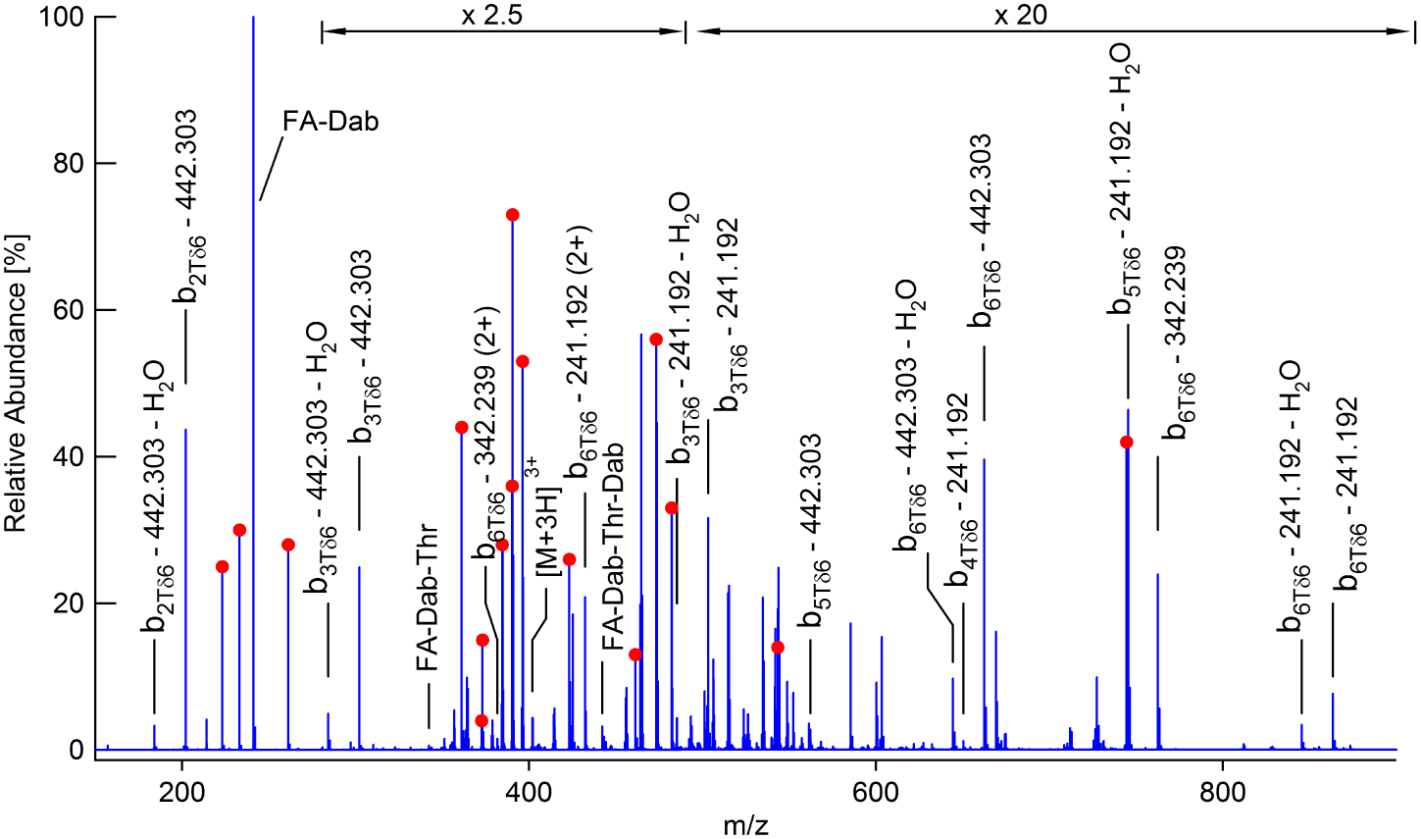
CID product ion spectrum for polymyxin extracted by taking a horizontal slice through the plot in Figure 5a at *m*/*z* 402. Peaks corresponding to initial ring cleavage at the Thr-Dab bond are explicitly annotated, other assigned peaks are indicated with a red dot.

Pittenauer et al.^51^ and Govaerts et al.^50^ have reported CID product ion spectra for the [M + H]^+^ and [M+2H]^2+^ polymyxin precursors, respectively. Nominal mass ion trap instruments employing precursor isolation windows were used in both cases. All 10 peptide bonds were cleaved, and a total of 26 peaks were assigned to sequence ions in the same *m*/*z* range as Figure 7. We find 19 of these peaks from the combined earlier studies and assign a further 12 peaks to sequence ions (including 7 doubly charged ions), i.e., a total of 31 assignments. Again, cleavages of all 10 peptide bonds are represented. Equivalent structural information has therefore been obtained by 2DMS, but the analysis has been performed in parallel with MS/MS analysis of 6 other precursors. Product ion spectra for each of these can also be extracted by taking horizontal slices through Figure 5a. The analyte is used efficiently in 2DMS (because there is no isolation window) and the prodigious peak capacity (resolving power in product axis x resolving power in the precursor axis = 4 × 10^6^) may allow simultaneous analysis of many more precursors.

In UVPD, photon absorption leads to excitation of the ion to a higher electronic state and thereafter to dissociation via statistical or nonstatistical pathways.^27,53^ The former favor the same low-energy, mobile proton routes to b and y fragments that are typical of slow-heating activation methods such as CID and IRMPD whereas the latter access reactions with higher activation barriers.^53^ These additional high energy reaction channels offer more coverage and consequently higher confidence when sequencing peptides. UVPD spectra often exhibit a, a+1, a+2, b, c, x, y, y-1, y-2, and z fragments from backbone cleavages, and d, w, and v fragments from side chain cleavages.

As an example, Figure 8 shows an annotated product ion spectrum corresponding to the melittin [M+5H]^5+^ precursor, which has been extracted from Figure 5b by taking a horizontal slice at *m*/*z* 571. Photodissociation is extensive and a rich spectrum of fragments with +1, + 2, and +3 charge states is observed. The [M+5H]^5+^ precursor population is heavily depleted (2% relative abundance) despite a total energy input of only 150 μJ. Beam CID was minimized by setting the collision energy to a low value (8 eV).

**Figure 8:**
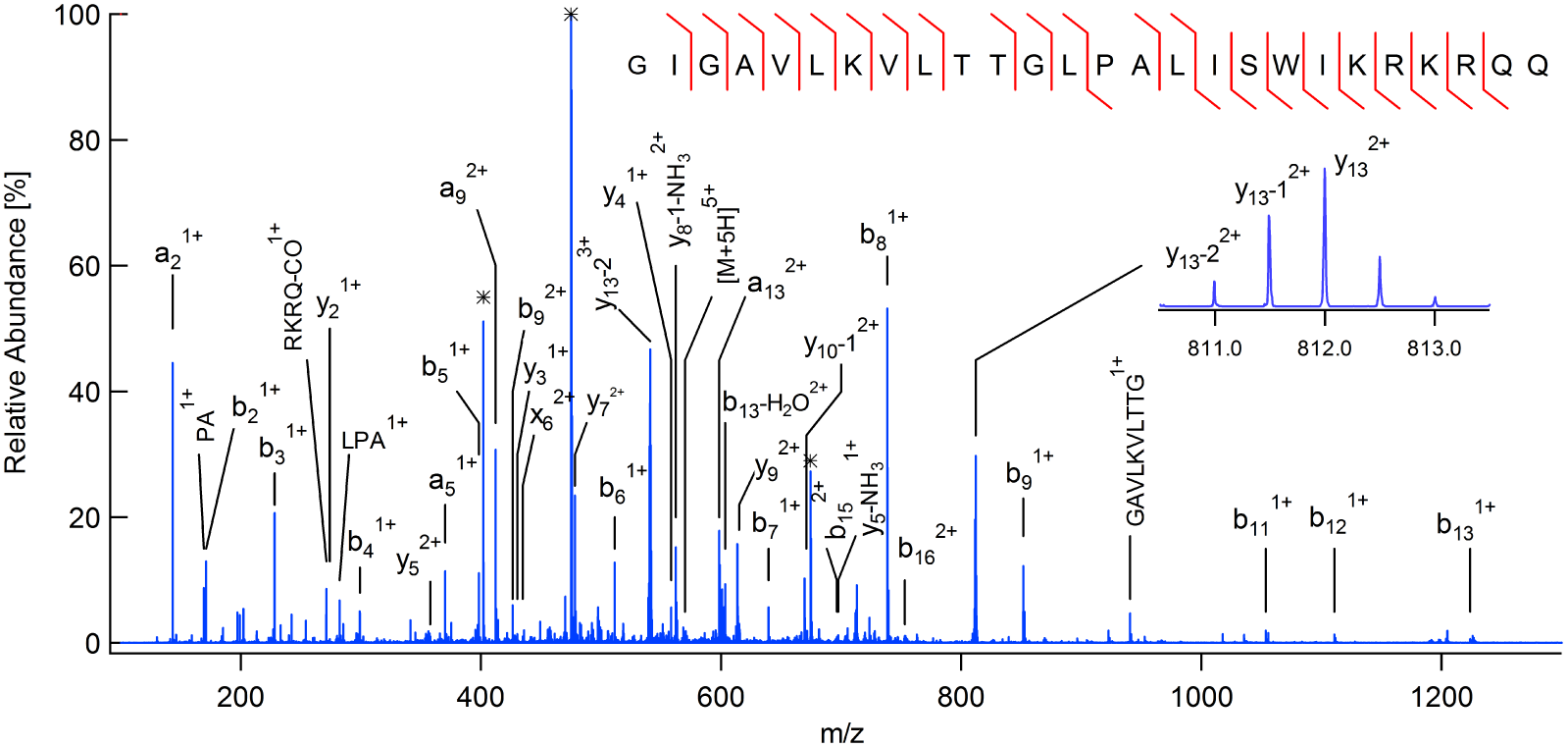
UVPD product ion spectrum for melittin extracted by taking a horizontal slice through the plot in Figure 5b at *m*/*z* 571. Artifacts arising from intersection of the slice with a vertical streak are indicated with an asterisk. Examples of UVPD-specific fragments are shown in the inset plot.

Three prominent peaks are artifacts attributable to the vertical streaking mentioned earlier. A horizontal slice through the data indiscriminately samples both genuine peaks and the high noise floor in any vertical slice containing an intense precursor peak. However, these artifacts may be readily eliminated (either manually or using automated peak-picking) by requiring genuine peaks to be local maxima with signal-to-noise ratios above a threshold value.

Only four backbone cleavages are missed, yielding a substantially better sequence coverage (84%) with UVPD than can be achieved using CID.^54^ There are several possible z fragments, but these are indistinguishable from y-NH_3_ fragments due to the presence of lysine, arginine, and glutamine residues at the C-terminus, all of which may lose ammonia. However, some UVPD-specific products can be unambiguously identified. For example, y_13_-1 and y_13_-2 fragments are prominent (see inset plot in Figure 8). The latter fragment is a specific characteristic of proline residues.^43^ Molecular dynamics simulations have shown that initial activation of C–C and C–N bonds close to proline followed by a series or rearrangements and proton transfers is the likely reaction pathway.^55^ Proline-directed cleavage is also well-known in CID and indeed a strong y_13_ peak is present in Figure 5a. However, the y_13_-1 and y_13_-2 fragments are absent as entirely different reaction mechanisms are involved. Substance P has two proline residues, and it too exhibits y_8_-2 and y_10_-2 fragments (not shown) by UVPD in common with previous work.^43^

## Conclusions

The SWIM encoding technique has been successfully applied to ions trapped in a linear quadrupole. Mixtures can be analyzed without chromatographic separation of the components by coupling the ion cloud radius modulation with fragmentation by CID or UVPD. At low excitation amplitudes, the flux-constant mode is dominant. At higher excitation amplitudes, there is a transition to the flux-periodic mode in which the population of transmitted ions is modulated.

Peaks in the 2D mass spectrum can be assigned with high confidence because the resolution and mass accuracy of the ToF analyzer is retained in the product ion axis. The annotated spectra shown in Figures 7 and 8 could have been obtained using traditional chromatographic separation and/or targeted precursor isolation. However, the advantages of 2DMS are now clear. The burden of operating a chromatography system has been entirely bypassed and prior knowledge of the mixture composition is not required.

In principle, other mass analyzers that may be coupled to a linear quadrupole ion trap, including the Orbitrap, could be used instead of a ToF analyzer.

## Supplementary Material



## Notes

Peak widths and mass accuracy in both *m*/*z* axes, additional simulations, simulation description and code listing (PDF)
