# Supplementary material for: Chromatography-Free Analysis of Mixtures Using a Two-Dimensional Mass Spectrometry (2DMS)-Enabled Quadrupole Time-of-Flight (QToF) Analyzer
Source: Anal Chem. 2026 Apr 23;98(17):12883–94. doi: 10.1021/acs.analchem.6c00486 (PMC13150812; doi:10.1021/acs.analchem.6c00486)
Supplement: Supplementary file 1 [file ac6c00486_si_001.pdf]

Chromatography-free analysis of mixtures using a two-dimensional mass spectrometry (2DMS)-enabled quadrupole time-of-flight (QToF) analyzer

Steven Wright,[1] Nathan Cassidy,[1] Alex Colburn,[1,2] and Peter B. O' Connor[*1,2]

[1] Verdel Instruments Ltd, 154k Brook Drive, Milton Park, Abingdon, Oxfordshire, OX14 4SD, UK

[2] Department of Chemistry, University of Warwick, Coventry, CV4 7AL, UK

* Corresponding author

SUPPORTING INFORMATION

Contents

1. Additional simulations....S2
2. Calibration of precursor axis S3
3. Precursor axis mass accuracy and peak widths....S4
4. Product axis mass accuracy and peak widths S5
5. Simulation strategy....S6
6. SIMION code listing for SWIM simulation....S9

1. Additional simulations

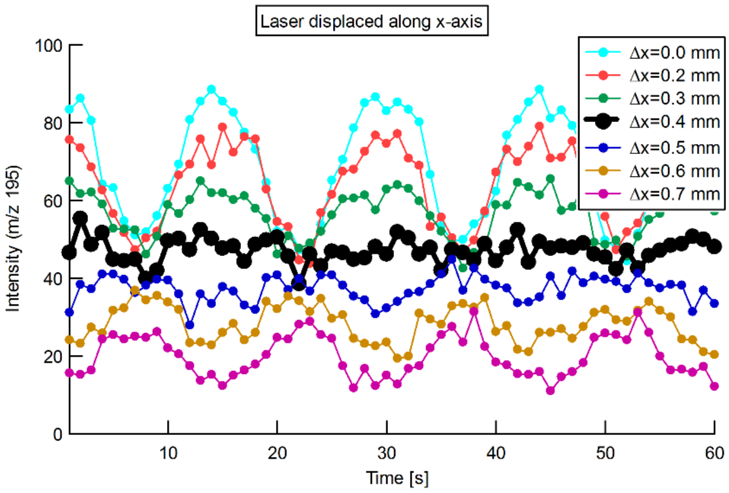

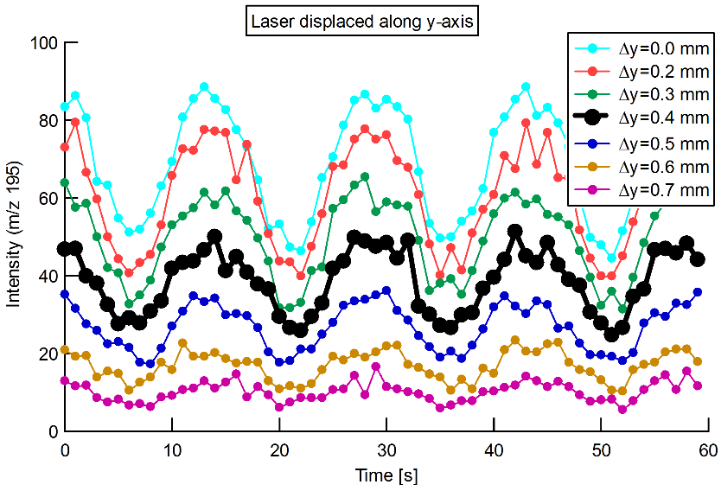

Fig. S1. Effect of laser beam displacement on product ion modulations in flux constant mode. Dipolar excitation is applied to the rod pair aligned with the x-axis and causes expansion of the ion cloud in the same direction. For x-axis displacements, the ion cloud elongates both towards and away from the laser beam centre during excitation, resulting in a null point (no overall modulation) at Δx≈0.4 mm and an inversion of the modulation phase at bigger displacements. Displacements in the y-axis move the elongated ion cloud symmetrically into a less intense region of the Gaussian laser beam, which reduces the fragment yield but maintains the same modulation profile.

2. Calibration of precursor axis

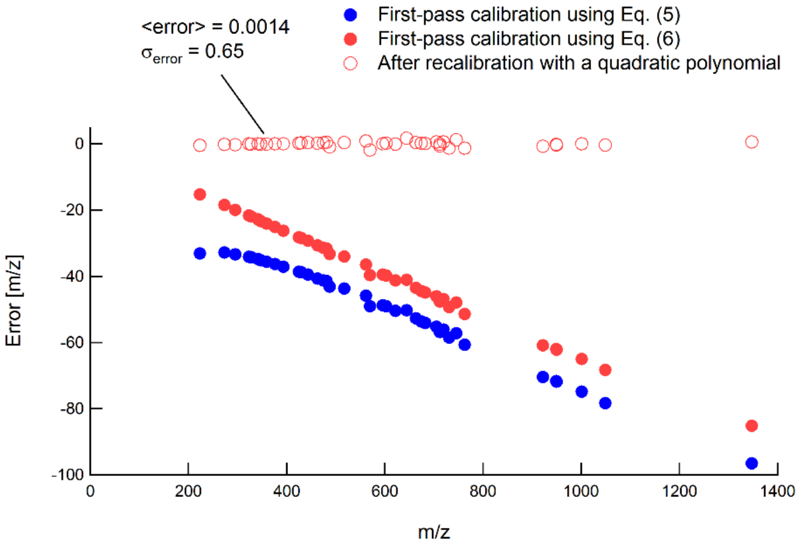

Fig. S2. Precursor axis calibration for Fig. 5(a) using peaks lying on the autocorrelation line. The error value is the difference between the peak position on the y-axis in the 2D spectrum and the known accurate mass provided by the x-coordinate. A first-pass calibration using either Eq. (5) or (6) converts from encoding frequency to *m/z*. A 5-7% error is typical, and primarily due to the difficulty in measuring V_{RF} accurately. Eq. (5) leads to a growing curvature below *m/z* 400 due to use of the Dehmelt approximation. Eq. (6) gives a more linear conversion and is a better starting point for subsequent removal of the residual error using a polynomial fit. The average error and standard deviation after a first pass calibration using Eq. 6 followed by a quadratic fit to the residual error are 0.0014 *m/z* and 0.65 *m/z*, respectively.

3. Precursor axis mass accuracy and peak widths

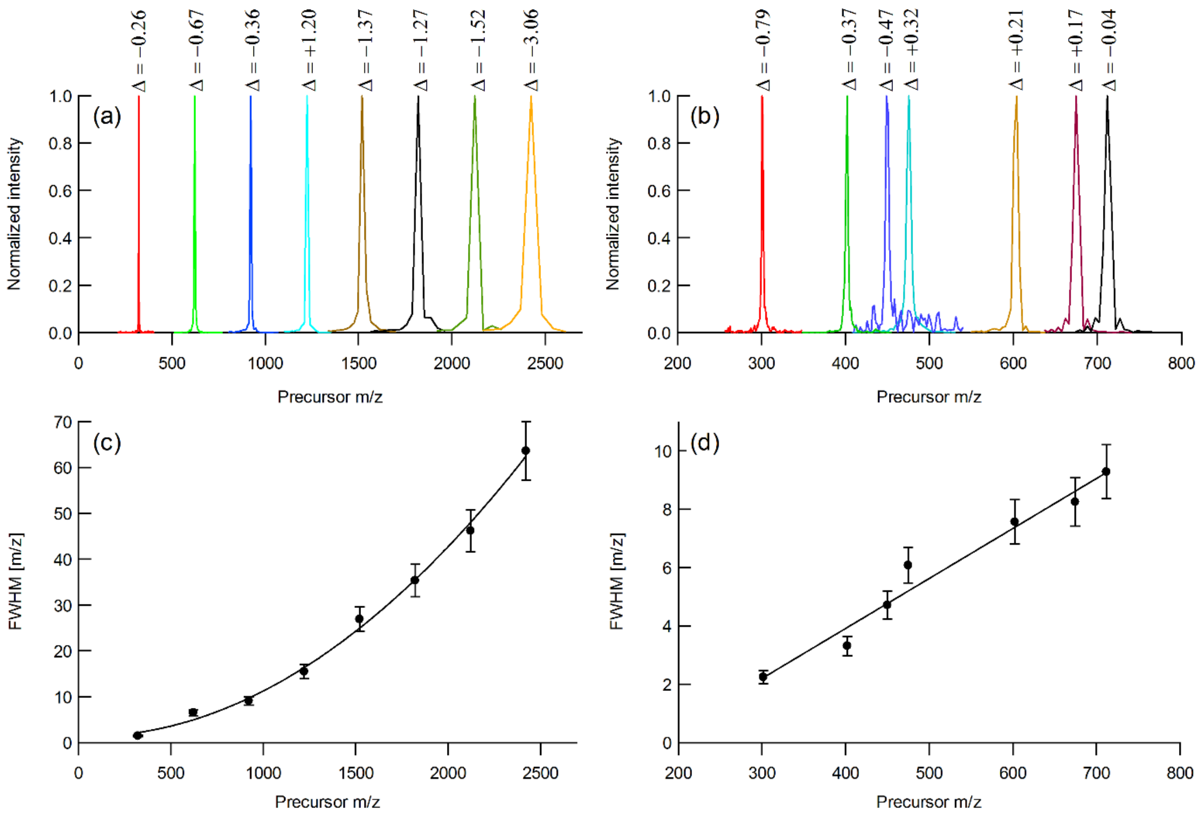

Fig. S3. Precursor peaks annotated with the *m/z* error of the centroid position after a first-pass calibration using Eq.6 followed by a polynomial fit for a) Agilent tune mix and b) peptide mix (data extracted from Figs 5(a) and (b)). Corresponding FWHM peak widths are shown in (c) and (d), respectively.

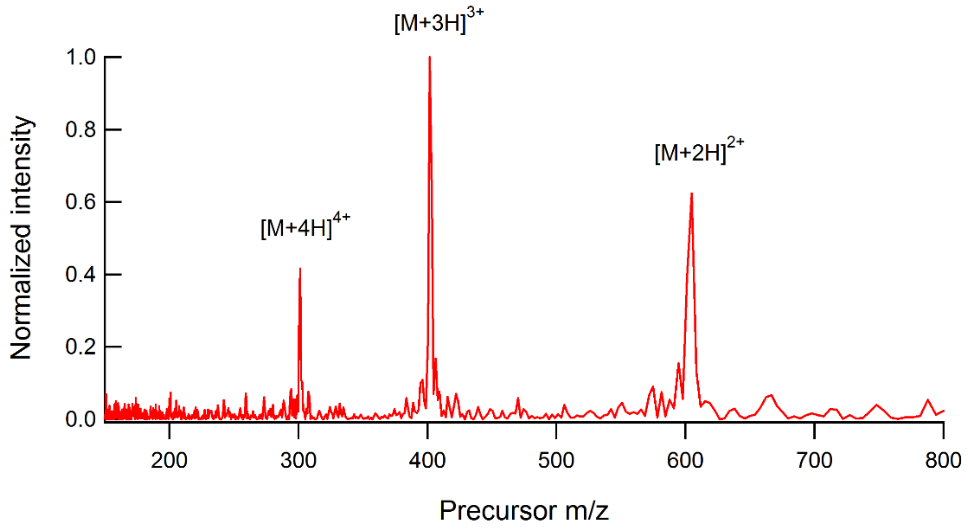

Fig. S4. Vertical slice through Fig. 5(b) at x=202.1189 *m/z*. This is a precursor scan showing that the $[T\text{-}DAB]^+$ product ion is common to all three polymyxin precursors. The peaks are well-resolved in this example.

4. Product axis mass accuracy and peak widths

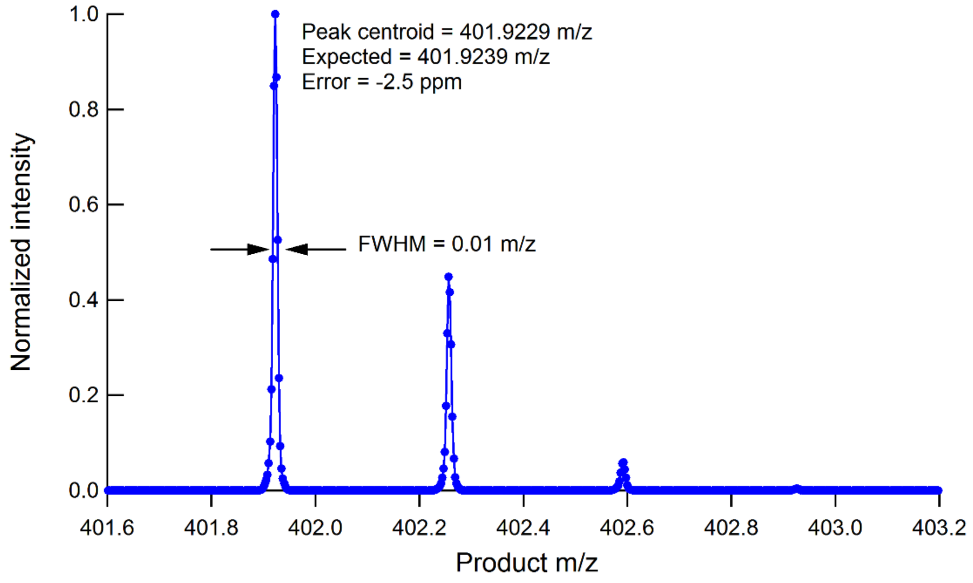

Fig. S5. Horizontal slice through Fig. 5(b) at y = 401.91 *m/z*. Section showing the polymyxin $[M+3H]^{3+}$ precursor with isotope peaks. The resolution and mass accuracy of the ToF analyser are retained in this dimension.

5. Simulation strategy

All simulations were performed using SIMION 8.2 installed on a server with 128 GB of RAM, a 1.8 TB hard drive, and an Intel Xeon Bronze 3104 CPU. Loading of pre-calculated SWIM pulse and ion definition files, generating time-varying voltages, and data extraction were all controlled using the embedded LUA programming application. Typically, a sequence of 140 SWIM pulses applied to a population of 1000 ions was simulated in 15 hrs using 8-10 of the 12 available physical cores.

A two-part simulation strategy was adopted. Firstly, an initial ion cloud was simulated using a full 3D model of the ion quide, lenses, and precision quadrupole using a relatively coarse scaling of 1 grid unit = 50 µm. Ions randomly generated in time and space within the ion guide were injected into the precision quadrupole, trapped and allowed to cool through collisions with background gas. Collisions were modelled using the HS2 hard-sphere model provided with SIMION and experimental collisional cross-sections.[1] After an elapsed cooling time of 1-8 ms, the *x*, *y*, *z* coordinates and corresponding velocity components of ions positioned within the precision quadrupole were saved to file. In the second stage of the simulation, these instantaneous ion positions and velocities were then transferred to a higher definition model of just the precision quadrupole for simulation of the SWIM excitation and UVPD, ensuring that the RF phase at the time of transfer was also preserved. The scaling was reduced to 1 grid unit = 1 µm and the hyperbolic field was defined as a 2D array in the (*x*,*y*) plane, extruded to +/- 60 mm in the *z*-axis.

The ion optical components of the Bruker MaXis II (ETD) QToF mass spectrometer (Bruker Daltonik GmbH, Bremen, Germany) were used as the basis for the geometric model. The quadrupole ion guide, prefilter, precision quadrupole, post filter and both lens stacks were all included but insulating components were not. The precision quadrupole has a field radius of r_0 = 3 mm and is fabricated as four truncated hyperbolic shim electrodes mounted on a glass envelope. This was modelled as four solid, free-floating hyperbolic rods with the same beam-facing profile.

At the start of the SWIM simulation, the initial ion cloud definition and pre-calculated SWIM pulses were read from file and stored in arrays. Typically, 1000 ions were used to simulate each trap-and-excite cycle, which were chosen randomly from a list of 5000 ions in the ion cloud definition array, generated in the first part of the simulation. The SWIM pulse amplitude was calculated at 40 ns time intervals and the simulation was performed with the same time step. However, an interpolation procedure must be provided within the *fast_adjust* LUA segment as the Runge-Kutta trajectory calculation makes multiple calls to this segment at intermediate times during each time step.

The ion trajectories were simulated one at a time and a tally was kept of the number of precursor and product ions in the trap at the end of the 20 ms cycle. As a simplification, all the photons are assumed to be delivered at t=13 ms in a single pulse with a pulse width shorter than the simulation time step. The fragmentation probability for a precursor ion at radius r at the instant of irradiation is $1-exp(-\sigma N_p P(r))$, where σ is the photofragmentation cross-section, N_p is the total number of photons in the pulse, and $P(r)$ is the normalized probability density function. However, using an averaged fragmentation probability for one orbit of the secular motion at the time of the laser pulse results in better statistics.

If the photon energy is greater than the dissociation energy of the bond broken during UVPD then the excess energy is partitioned between translational energy and excitation of internal modes. To include this additional source of translational energy in the simulations, the amount of excess energy distributed between the fragment translational degrees of freedom, E_T, was included as a variable. Noting that the photon energy at 213 nm is 5.8 *eV* while typical bond energies lie in the range of 3-4 *eV*, E_T =1 eV was used as an estimate. Energy and momentum balance require that the magnitude of the center-of-mass velocity of a fragment A is $u_A=(2m_BE_T/m_Am_{AB})^{0.5}$, where m_A, m_B, and m_{AB}, are the masses of the two fragments and precursor, respectively. Although photofragmentation product angular distributions may be peaked, they are frequently anisotropic, particularly when the fragmentation process is slow compared with rotation.[2] We assume the latter and generate uniformly distributed fragment ion

velocity vectors in the center-of-mass frame. In the laboratory frame, the velocity of the fragment ion is the vector sum of the precursor velocity and the fragment center-of-mass velocity.

References

(1) Yavor, M. I.; Shcherbakov, A. P.; Pomozov, T. V.; Kirillov, S. N.; Vorobjev, A. N.; Makarov, V. V.; Verenchikov, A. N. Axial and Radial Space-Charge Effects in Radiofrequency Gas-Filled Low-Pressure Quadrupole Ion Guides. *Int. J. Mass Spectrom.* **2023**, *491*, 117097. https://doi.org/10.1016/j.ijms.2023.117097.

(2) Lefebvre-Brion, H.; Field, R. W. Photodissociation Dynamics. In *The Spectra and Dynamics of Diatomic Molecules*; Elsevier, 2004; pp 469–549. https://doi.org/10.1016/B978-012441455-6/50010-X.

6. SIMION code listing for SWIM simulation

```
--[[ Simulation of SWIM excitation using an initial ion cloud distribution. SWIM pulses are pre-
calculated and stored as csv files.--]]

adjustable RF_amp = 120.0            -- RF amplitude (0-pk)
adjustable RF_freq = 1.02e6          -- RF frequency (Hz)
adjustable scale_SWIM = 387          -- scaling of SWIM pulse
adjustable beam_rad = 0.8            -- apertured laser radius(mm)
adjustable sigma=0.26                -- sigma of laser beam profile (mm)
adjustable neutral_mass = 414        -- neutral lost in Da
adjustable eV_avail=1.0              -- fraction of E0-hv in eV
adjustable orbit_av_beg = 13000
adjustable orbit_av_end = 13013
adjustable delta_t = 0.04            -- simulation time step (us)
adjustable cent_offset = 10          -- x axis offset to match gem file (mm)
adjustable frag_xsection = 4e-15     -- fragmentation cross-section (mm^-2)
adjustable photons=1.2e14            -- no of photons in laser pulse
adjustable z_ext=60                  -- z axis extends to +/- z_ext (mm)


adjustable start_file=113            -- first SWIM index
adjustable end_file=129              -- last SWIM index

local SWIM_duration
local initial_mass                   -- precursor mass
local SWIM ={}
local max_index                             -- points in SWIM file
local max_ions                       -- no of initial ions available
local all_frags, all_precursors
local results                        -- output precursors and fragments
local period                         -- rf period
local num_of_steps                   -- steps per rf period
local path_sum=0                            -- sum fragment probability one orbit


local xp={}                          -- arrays for input ion params
local xv={}
local yp={}
local yv={}
local zp={}
local zv={}

--Import collision model and over-write variables.

local HS1 = simion.import("collision_hs1.lua")

adjustable _temperature_k      = 293.0-- Background gas temperature (K)
adjustable _sigma_m2           = 2.25E-18       -- Collision-cross section (m^2)
adjustable _gas_mass_amu       = 28.0  -- Mass of background gas
adjustable _mark_collisions    = 1              -- 1=yes,0=no
adjustable _pressure_pa        = 0.005-- Background gas pressure (Pa)

function gaussian_random()
-- Gaussian distribution needed for photofragmentation events
-- Using the Box-Muller algorithm. Copied from collision_hs1.lua
   local s = 1
```

```
    local v1, v2
    while s >= 1 do
        v1 = 2*rand() - 1
        v2 = 2*rand() - 1
        s = v1*v1 + v2*v2
    end
    local g_rand = v1*sqrt(-2*ln(s) / s)
    return g_rand
end

function photo(old_vx,old_vy,old_vz)
      --changes velocity of charged fragment after photofragmentation

      local dalton=1.67e-27
      local E_avail=eV_avail*1.6e-19
      local mA=(initial_mass-neutral_mass)*dalton
      local mB=neutral_mass*dalton
      local mAB=initial_mass*dalton
      local uA=0.001*sqrt(2*E_avail*mB/(mA*mAB))
      local ux=gaussian_random()
      local uy=gaussian_random()
      local uz=gaussian_random()
      local norm=1/sqrt(ux^2+uy^2+uz^2)
      ux=uA*norm*ux
      uy=uA*norm*uy
      uz=uA*norm*uz
      return old_vx+ux, old_vy+uy, old_vz+uz
end

function segment.flym()

            results = assert(io.open("results_"..start_file..".csv","w"))

            --load ion parameters from file to local arrays
            local     pxx,vxx,pyy,vyy,pzz,vzz,notused
            local n=1
            for line in io.lines("ion_log.csv") do
                        pxx,vxx,pyy,vyy,pzz,vzz,notused=line:match("%s*(.-),%s*(.-),%s*(.-),%s*(.-),%s*(.-
),%s*(.-),%s*(.*)")
                        xp[n]=tonumber(pxx)+cent_offset
                        xv[n]=tonumber(vxx)
                        yp[n]=tonumber(pyy)
                        yv[n]=tonumber(vyy)
                        zp[n]=tonumber(pzz)
                        zv[n]=tonumber(vzz)
                        n=n+1
            end
            max_ions=table.getn(xp)
            print(max_ions)
            --Coerce period to integer x delta_t
            period = 1e6/RF_freq
            num_of_steps = math.floor((period/delta_t)+0.5)
            period = num_of_steps*delta_t
            RF_freq = 1e6/period

            --Warnings splash screen
            print("Frequency coerced to "..RF_freq.. " Hz")
```

```
	--programmatically set trajectory retain off and Tqual=0
	sim_trajectory_image_control=1
	sim_trajectory_quality=0

	--iterate through all the allocated SWIM files
	for file_num=start_file,end_file do
		local file_stem, new_file_name
		file_stem="SWIM/x-SWIM-Pulse.csv"
		new_file_name=string.gsub(file_stem, "x", file_num)

		--load SWIM csv file
		local i=0
		local time, volts
	for line in io.lines(new_file_name) do
		if i~=0 then
			time,volts = line:match("%s*(.-),%s*(.*)")
			SWIM[i] = tonumber(volts)*scale_SWIM
			end
		i=i+1
	end

		--run simulation for current SWIM file
		max_index=table.getn(SWIM)
		SWIM_duration = delta_t * max_index
		all_frags=0
		all_precursors=0
		--load_ions:seek("set")
		run()
		print (file_num,all_precursors,all_frags)
		if file_num == 1 then
			--write header info to results file
			results:write("RF_frequency=", RF_freq, "\n")
			results:write("RF_amp=", RF_amp, "\n")
			results:write("scale_SWIM=", scale_SWIM, "\n")
			results:write("neutral_mass=", neutral_mass, "\n")
			results:write("frag_xsection=", frag_xsection, "\n")
			results:write("photons=", photons, "\n")
			results:write("initial_mass=", initial_mass, "\n")
			results:write("beam_rad=", beam_rad, "\n")
			results:write("sigma=", sigma, "\n")
		end
		results:write(file_num,",",all_precursors,",",all_frags,"\n")
		results:flush()
	end
	results:close()
end

function segment.initialize()

	-- randomly select ion from array

	local m=math.random(1, max_ions)
	ion_px_mm=xp[m]
	ion_vx_mm=xv[m]
	ion_py_mm=yp[m]
	ion_vy_mm=yv[m]
```

```
        ion_pz_mm=zp[m]
        ion_vz_mm=zv[m]
        initial_mass = ion_mass
end

function segment.fast_adjust()

        local omega = RF_freq * (1E-6 * 2 * math.pi)
        local V_trap = RF_amp * sin(ion_time_of_flight * omega)
        local V_aux = 0.0
        local index
        local fraction

        -- Interpolate between array points if necessary
        index = math.floor((ion_time_of_flight)/delta_t)
        fraction=ion_time_of_flight/delta_t-index
        V_aux = SWIM[index+1]+fraction*(SWIM[index+2]- SWIM[index+1])

        adj_elect01 = V_trap
        adj_elect02 = -1*V_trap + V_aux
        adj_elect03 = -1*V_trap - V_aux

end

function segment.tstep_adjust()

        ion_time_step = delta_t
end

function segment.other_actions()

        local density_func              --Gaussian probability density function
        local av_frag_prob              --averaged probability

        -- Invoke collision model
        HS1.segment.other_actions()

        -- Ion about to leave volume is returned through opposite face
        if ion_pz_mm+ion_vz_mm*delta_t>=z_ext then
                ion_pz_mm=ion_pz_mm-2*z_ext
        end
        if ion_pz_mm+ion_vz_mm*delta_t<=-z_ext then
                ion_pz_mm=ion_pz_mm+2*z_ext
        end
        -- Determines if ion fragments in laser
        -- (i)Average fragmentation probability for one secular period

    if ion_time_of_flight > orbit_av_beg
        and
        ion_time_of_flight <= orbit_av_end
        then
                local r, az= rect_to_polar(ion_px_mm-cent_offset, ion_py_mm)
                if r < beam_rad then
                density_func=(1/(2*math.pi*sigma^2))*exp(-(r^2)/(2*sigma^2))
                path_sum=path_sum+1-exp(-frag_xsection*photons*density_func)
                end
        end
```

```
        --(ii) Determine if ion fragments using Monte Carlo
        if ion_time_of_flight > orbit_av_end
        and
        ion_time_of_flight <= orbit_av_end + delta_t
        then
                av_frag_prob=path_sum*delta_t/(orbit_av_end-orbit_av_beg)
                if av_frag_prob > rand() and ion_mass == initial_mass then
                        ion_mass = ion_mass - neutral_mass
                        ion_color = ion_color +1
                        ion_vx_mm,ion_vy_mm,ion_vz_mm=
                        photo(ion_vx_mm,ion_vy_mm,ion_vz_mm)
                end
        end

        --Keep tally of surviving precursors and fragments
        if ion_time_of_flight >= SWIM_duration-2*delta_t then
                ion_splat = -4
                if ion_mass == initial_mass then
                        all_precursors = all_precursors + 1
                else
                        all_frags = all_frags + 1
                end
        end

        --Reset summation when ion trajectory has ended
        if ion_splat == -1 or ion_splat == -3 or ion_splat == -4 then
                path_sum=0
        end

        --Error trap
        if ion_splat == -3 then
                print ("Boundary crossing error")
        end
end

function segment.terminate()
end
```
